# Effects of Consumers’ Sensory Attributes Perception on Their Willingness to Pay for Apple Cultivars Grown at Different Altitudes: Are They Different?

**DOI:** 10.3390/foods11193022

**Published:** 2022-09-29

**Authors:** Azucena Gracia, Celia M. Cantín

**Affiliations:** 1 Unidad de Economía Agroalimentaria, Centro de Investigación y Tecnología Agroalimentaria de Aragón (CITA), Avda. Montañana, 930, 50059 Zaragoza, Spain; 2 Instituto Agroalimentario de Aragón–IA2, CITA-Universidad de Zaragoza, 50059 Zaragoza, Spain; 3 Departamento de Pomología, Estación Experimental de Aula Dei (EEAD-CSIC), Avda. Montañana, 1005, 50059 Zaragoza, Spain

**Keywords:** auction, consumer acceptance, hedonic evaluation, preferences

## Abstract

Per capita apple consumption is decreasing in many European countries due to the perception that apples have inferior sensory traits and to the dissatisfaction of some consumers with mainstream apple cultivars. Previous studies on the consumers’ acceptance of apples state that sensory attributes have a large influence on consumers’ willingness to pay (WTP). Following this previous evidence and with the aim of reversing this decreasing consumption trend, our objective was to study the effect of apple sensory attributes on consumers’ WTP for a mainstream (‘Golden Delicious’) and a local traditional apple cultivar (‘Reineta’), grown at two different altitudes (high and low). A total number of 195 apple consumers participated in one experiment with two tasks. In the first task, participants were asked to rate five external and internal sensory attributes, and in the second, to indicate their maximum WTP in an experimental auction for the four apple samples. Results indicated that the WTP and the effect of sensory attributes on the WTP were different among the four apple samples. Our findings indicate that sweeter and juicer apples might be marketed at higher prices and that firmness is only appreciated in the case of the local cultivar grown at low altitudes.

## 1. Introduction

Per capita apple consumption is decreasing in many European countries [1], and this is the case in Spain. The reasons for this decline, apart from the economic factors such as incomes and prices and consumers’ demographic characteristics, are related to the apples’ sensory attributes and to their substitution by other fruits. Regarding the reasons related to the apple’s sensory traits, the main causes for the reduction in their consumption are those related to the perception that apples have inferior sensory characteristics (e.g., less tasty) than in the past and also to the dissatisfaction of some consumers with available mainstream apple cultivars (such as ‘Golden Delicious’). Another reason to justify this consumption decline is the competition with a wider range of other fruits available from which to choose than in the past and the perception that some of them are healthier than apples [1]. In this context, the ability of producers and marketers to respond to consumers’ sensory expectations and the dissatisfaction with mainstream apple quality will be very important to reverse this negative consumption trend. Therefore, providing a better understanding of consumers’ preferences, sensory perceptions, and dissatisfaction is crucial for producers and marketers to increase the demand for apples.

Several studies on consumers’ perception and acceptance of apples identified that sensory quality (flavor, taste, and texture) is one of the most important factors influencing the decision to buy apples [2], even more important than the price [3]. Food product quality has both an objective and a subjective dimension. Objective quality refers to the physical characteristics of the food product and is typically measured by engineers and food technologists using instrumentation and physical and chemical techniques. Otherwise, subjective quality is the quality as perceived by consumers and is measured using consumers’ sensory attributes evaluation tests [4,5]. The instrumental measurements are only able to capture one aspect of food, and although it is essential for routine quality control of the products, this may not always be sufficient to determine whether the quality of the product is to be accepted in the market. On the other hand, the sensory evaluation gives a judgment of the overall acceptability of a product because only consumers can determine whether a new product is acceptable [5]. Taking this into account, some empirical papers have analyzed consumers’ apples acceptance using consumers’ sensory evaluations and studying their relationship with the consumers’ willingness to pay (WTP) [6,7,8,9,10]. They studied the effect of external and internal sensory attributes (e.g., firmness, sweetness) on consumers’ WTP for different apple cultivars. Although these studies differ in the way they measure the WTP (contingent valuation, auctions), internal sensory attributes are reported to influence the premium price that consumers are willing to pay in all the studies, whereas external appearance is an influential factor in some of them. These papers state that apples perceived superior in texture (e.g., firmness) and in flavor (e.g., sweetness) would receive higher consumers’ WTP. However, the impact of consumers’ socio-demographic characteristics, such as age, ethnicity, and employment, was not so important, and only a few of them explain the WTP for apples, and always to a lesser extent than in the case of the sensory attributes.

Some of them also studied whether the effect of the sensory attributes assessment on the WTP is different among cultivars using different assumptions [6,7,8,10]. In general, they found that sensory attributes are key to the WTP but that the effect of those attributes on the WTPs differs among apple cultivars. For instance, Hong et al. [6] studied the effect of external appearance, firmness, juiciness, and sweetness on the WTP for ‘Delicia’, ‘Royal Gala’, and ‘Fuji’ apples and found that, while firmness positively influenced the WTP for ‘Royal Gala’, this quality trait did not impact the WTP for ‘Delicia’ and ‘Fuji’. In addition, juiciness had a negative impact on the WTP for ‘Delicia’, whereas this impact was positive for ‘Royal Gala’, and no effect was observed for ‘Fuji’.

In addition, previous papers have already demonstrated the impact of growing altitude on apple sensory attributes perception by consumers [11,12,13]. However, as far as we know, no previous papers have investigated the differences in consumers’ WTP and the effect of the sensory attributes’ perception on the WTP for apples grown at different altitudes. Following this previous evidence, our objective was to study the effect of apple sensory attributes perception on consumers’ WTP for apples grown at two different altitudes (high and low). Two different apple cultivars were chosen for this work in order to investigate differences in the consumer response between a mainstream, well-known, and highly consumed and produced cultivar (‘Golden Delicious’) versus a traditional cultivar from the region where the experiment was conducted (‘Reineta’). Results from this comparison will be very useful for local producers and distributors when designing marketing strategies. By combining data from an experimental auction with sensory evaluation from untrained consumers evaluations, we tested three research hypotheses: (i) the WTP and the sensory attributes consumers’ perception differ among apple cultivars and growing altitudes; (ii) the effect of sensory attributes on these WTP also differs among apple cultivars and growing altitudes; (iii) consumers’ socio-demographic characteristics influence the WTP for the different apple samples. Finally, all these effects on the WTP were measured, and implications for the apple industry are discussed.

## 2. Materials and Methods

### 2.1. Products

**Figure 1:**
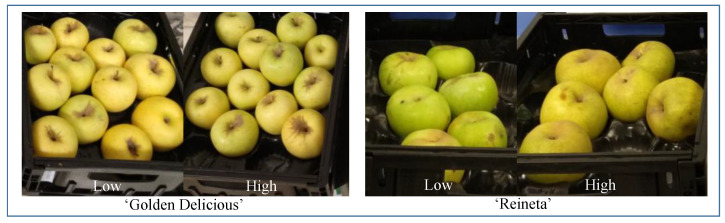
‘Golden Delicious.’ and ‘Reineta’ apple cultivars grown at low and high altitudes provided to the consumers in the consumer study.

Two apple cultivars, a widespread mainstream cultivar (‘Golden Delicious’) and a local traditional (‘Reineta’) apple, were selected, and both were produced at both high and low altitudes. For the low altitude, apples were cultivated in a valley site (Ebro valley, Zaragoza, ca. 300 m a.s.l.); and for the higher altitude, apples were cultivated in a hilly site (Manubles valley, Moros, ca. 800 m a.s.l.) (Figure 1). The ‘Golden D.’ from the Ebro valley were purchased from a cooperative located at “La Almunia”, around 45 km from Zaragoza, and the ‘Reineta’ fruit came from the experimental plots at the Centro de Investigación y Tecnología Agroalimentaria de Aragón (CITA). Both apples from the hilly site (Manubles Valley) were purchased from the fruit cooperative located in the mountain village of Moros. The apples were kept in cold storage (1.5 °C under a normal atmosphere and 80% relative humidity (RH)) until the evaluations (4 and 6 weeks of cold storage for ‘Reineta’ and ‘Golden D.’, respectively). After storage, fruits were visually selected in order to provide a homogeneous fruit sample to consumers. Then, we studied four apple samples: ‘Golden D.’ and ‘Reineta’ cultivated at high and low altitudes.

### 2.2. Participants

Participants were recruited by the research team with the help of consumers’ associations and public institutions in different locations (universities, town hall learning centers, community activity centers, etc.) through personal on-site contact, posters, and e-mail posting lists. The consumer study was performed at various places in Zaragoza (middle-sized town in the Northeast of Spain close to the two growing sites). The target population was primary food shoppers of the household who consume apples at home and older than 18 years. They received a gift card with a value of EUR 20 to take lunch/dinner in a pre-established restaurant located in the city in the next 4 weeks.

### 2.3. Experimental Procedure

The experiment was conducted in 20 working sessions of around 10 participants, reaching a total number of 195 participants. Each session lasted approximately 1 h and was carried out either in the morning, mid-day, afternoon, or evening. The evening times were chosen to allow working people to be able to take part in the study. The Ethical Committee of CITA approved the experiment protocol. All participants voluntarily joined the experiment, and previously to the session, they received information on the nature of the experiment and signed an informed consent form of participation. To keep anonymity, each participant was assigned an identification number upon arrival. The experiment was conducted from October to November 2019, when the apples were in season.

The working session consisted of three tasks: a consumer evaluation test and the experimental auction, followed by the fulfillment of a brief questionnaire. The same monitor explained the general instructions for the sessions and guided participants to complete the different tasks. The same two monitors conducted all the working sessions. First, participants received information about both apple’ cultivars and both growing sites. We provided this information before the sensory evaluation and the experimental auction because we wanted to mimic a consumption and re-purchase situation. In the whole purchase process, the first step is the first purchase moment, where consumers can observe the products on the shelves, and they obtain information from the external appearance and some extrinsic attributes (e.g., where the product was produced, the cultivar, etc.). Once at home, they consume the product and therefore obtain experience with the internal sensory attributes. This stage corresponds to our consumer evaluation of internal sensory attributes. Based on the experience gained in both stages (purchase and consumption at home), in a subsequent purchase situation, they would decide whether to re-purchase the product or not. Subsequent re-purchase is ultimately influenced by whether consumers liked the sensory attributes of the apples and their whole experience of eating the fruit [7]. In our case, given that participants received information about the extrinsic attributes, observed the appearance of the apples, and tasted them before the experimental auction, these auctions mimicked the re-purchase stage. Then, we expected to obtain a higher WTP than in the case in which participants would not have received any information and would not have gotten any experience from the tasting [14]. Indeed, our objective was to obtain the value for the apples in repetitive purchases, not in the first purchase.

Second, each participant could inspect both ‘Golden D.’ apples (high and low altitude) in a tray. Third, participants received two apple slices of this cultivar (‘Golden D.’), one of each growing altitude (high and low). They were asked to rate the overall external appearance and four textural (firmness, juiciness, mealiness) and flavor (sweetness) sensory attributes on a 9-point hedonic scale (1 = dislike extremely; 9 = like extremely). The descriptors were developed by consensus by the authors considering their experience and previous literature on apple sensory analysis. At the beginning of each session, the monitor sessions explained the descriptors to the volunteers and answered any doubts about the traits and the whole test they may have. The monitor also explained the difference between the sensory traits (objective) and the hedonic trait (subjective) they had to rate. The presentation order of the two ‘Golden D.’ apple samples (high and low growing altitude) was randomized to avoid ordering effects. Participants were instructed to cleanse their mouths between each tasting using water and/or crackers that were available on the tables.

After these ratings, the experimental auction was applied. The experimental auction is a procedure to measure consumers’ preferences in monetary terms (willingness to pay, WTP) by creating real and active market environments in which participants bid to buy the products through a mechanism that provides incentives for people to truthfully reveal the value they place on each of the products [15].

Some of the advantages of the auctions are that, in principle, they are not hypothetical because they involve exchanging real money for real goods and can directly provide each respondent with WTP values from the bids, unlike the choice experiment that needs to estimate the WTPs using discrete choice models [16]. Therefore, in our case, we used an experimental auction. However, because in the year of the experiment, the harvest of apples in the high-altitude area was almost inexistent (several long frozen nights when the trees were in full bloom), we could not have enough apples from the high altitude in order to exchange these apple samples (‘Golden D.’ and ‘Reineta’) for real money. We received some apple fruits for the sensory evaluation but not enough for their exchange in the auction for all the participants. Then, a hypothetical auction was finally conducted, and therefore, we can expect some hypothetical bias. In addition, the effectiveness of experimental auctions depends heavily on the choice of the auction mechanism [17]. The Vickrey auction (2nd price auction) mechanism is the most popular, followed by the Becker–DeGroot–Marschak (BDM). Far behind, we can find the Random nth price, although this mechanism overcomes some of the disadvantages of these two popular mechanisms (i.e., competitive bias, obtaining the most accurate data) [16]. However, it has the disadvantage of being difficult to explain to participants, difficult to logistically organize, and has a higher cost of implementation. In the last years, the Multiple Pricing List (MPL) mechanism has been increasingly used since it is easy to explain and understand for consumers [18]. Vickrey and Random nth price auctions require all participants to bid simultaneously, and the highest bidder wins and only has to pay the second (nth) highest price. In contrast, the BDM and MPL auctions do not require group participation, and it is suitable for individual experiments, which provides the assurance of randomness in sampling, as well as the avoidance of the information association defect caused by group auctions [17]. In our case, although we planned to conduct, in most cases, the experiment in group sessions, we allowed the use of individual or small group sessions to be able to stratify the sample according to the population’s needs. Therefore, we were forced to use an auction mechanism that could be applied in individual experiments. Finally, we decided to use the BDM since several papers proved that the BDM and the MPL mechanisms provide WTP estimates that do not differ significantly [18,19], but the BDM gives continuous response values instead of interval responses (MPL mechanism). In other words, the BDM provides continuous, detailed information about the individuals’ WTPs presenting higher variability to allow estimating the effect of apple sensory evaluation on consumers’ WTPs which is the aim of our study. In addition, the BDM method is able to map consumers’ preferences even for individuals with relatively low willingness to pay.

In a BDM auction, each participant submits a bid for a product; in particular, they indicate their maximum WTP for each auctioned product without knowing each other’s bids [18]. In our case, participants were asked for the maximum price they would be willing to pay for one kilo of each of the apple samples. Respondents should indicate the maximum price they are willing to pay just after each sample sensory evaluation. Because of the hypothetical nature of our experiment, at the end of the session, there was no exchange of apples for money.

**Figure 2:**
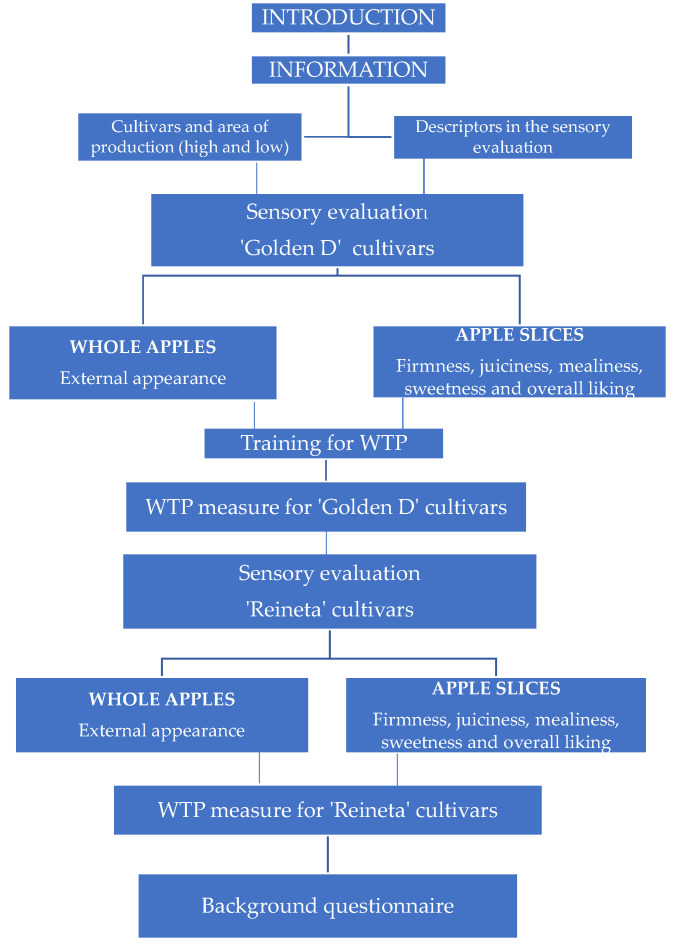
Flowchart of the experiment.

In the second task, participants could inspect both ‘Reineta’ apple samples (from high and low altitudes) in a tray, and a slice of each sample was also rated for the same descriptors, and the same question on the maximum price they would be willing to pay was asked. Finally, the participants were asked to fill in a questionnaire gathering information on economic and socio-demographic characteristics (age, gender, education, income, size, and composition of the household and province of residence), as well as fruit and apple purchasing and consumption habits. Figure 2 shows a flowchart of the experiment.

### 2.4. Statistical Analyses

First, differences in WTP and sensory attribute scores between growing sites (high and low altitude) for the same cultivar for all the participants were checked. In addition, differences in the WTP and sensory attribute scores between cultivars for the same altitude were tested. Therefore, paired *t*-tests, which are inferential statistics used to determine if there is a statistically significant difference between the means of two variables, were performed. In a second step, in order to test whether the effect of sensory traits on the WTP differs among apple cultivars and growing altitudes, we pooled the WTP information for the four apple samples, and we modeled this new WTP variable as a function of the sensory attributes and different dummy variables indicating each of the four apple samples. We defined three dummy variables (one was dropped to avoid multicollinearity) as follows: variable DGolHigh takes value 1 for the ‘Golden D.’ apple cultivated at high altitude and 0 otherwise; DGolLow takes the value 1 for the ‘Golden D.’ apple cultivated in low altitude and 0 otherwise, and DReiHigh takes the value 1 for the ‘Reineta’ apple cultivated in high altitude and 0 otherwise. A covariance analysis allows us to test whether the effect of the sensory attributes on the WTP is statistically different across apple samples. To do so, we used a Likelihood Ratio test (LR) between the whole model specified as a function of the sensory attributes, the new dummy variables, and the new dummy variables interacting with the sensory attributes and the restricted model (assuming that all the coefficients for the apple dummy variables and these variables interacting with the sensory attributes are equal to zero).

The *LR* is defined as follows:

(1) LR=−2(LL(0)−LL(1))

where *LL*(0) is the Log Likelihood under the null hypothesis, and the *LL*(1) is the Log Likelihood for the alternative hypothesis, in other words, for the restricted and the whole models, respectively.

If the null hypothesis where all the coefficients for the dummy apple variables and the dummies interacting with the sensory attributes is not rejected, we can conclude that the effect of sensory attributes on the WTP does not differ among the four apple samples. On the contrary, if the null hypothesis where all the coefficients for these dummy apple variables and the dummies interacting with the sensory attributes is rejected, then it indicates that the effect of sensory attributes on the WTP differs among the four apple samples. In the latter case, we should further investigate whether differences in the coefficient of the dummy variables and/or the dummy variables interacting with the sensory attributes exist using the LR test between the whole model and the appropriate restricted models. If the different restricted models are rejected, we can conclude that the effect of sensory attributes on the WTP totally differs among the four apple samples. In this case, we should estimate the relationships between the WTP and the sensory attributes separately for each of the four apple samples.

The WTP from a BDM auction is typically analyzed using Tobit models [15]. In our case, a left censored Tobit model is specified because, as observed in Table 1, sometimes participants gave zero values, and then the data are left censored. In censored models, the latent unobserved variable WTP* can be represented by the *WTP* really observed. We specified our *WTP* left censored as follows [20]:

(2) $$WTP^{*}=X_{i}\beta +\varepsilon_{ij}$$

$$WTP=0\;if\;WTP_{i}^{*}\;\leq 0$$

$$WTP=WTP_{i}^{*}\;if\;WTP_{i}^{*}>0$$

where *X_i_* is the vector of explanatory variables for individual *i*’s (overall external appearance, firmness, juiciness, mealiness, and sweetness), and *ε_ij_* is the error term assumed to be normally distributed with mean 0 and variance *σ*^2^. The parameter estimates are obtained by maximizing the likelihood function (*L*), which is represented by

(3) $$L=\prod_{i=1}^{N}(\frac{1}{\sigma }\phi {\left(\frac{WTP_{i}-X_{i}\beta }{\sigma }\right)}^{UC_{i}})\;\phi {\left(\frac{-X_{i}\beta }{\sigma }\right)}^{LC_{i}}$$

where *UC_i_* and *LC_i_* are indicator variables representing uncensored and left-censored *WTP*s, and *Φ* represents the cumulative standard normal distribution [15].

## 3. Results and Discussions

Around 60 percent of the respondents were women with an average age of 51 years (Table 1), and around 20% were in each of the age ranges (18–44, 45–54, 55–64, and over 65 years). Regarding education, 47% of the respondents had a university degree, and around 26% of them had primary or secondary studies. The average family size was three members, and 70% of households had no kids younger than 18. Only around 6% of participants stated to be vegetarian and eat 2.6 pieces of fruit daily. The consumers’ sample, as expected, consisted mainly of primary food shoppers in the household since around 80% of respondents did the grocery shopping always or often.

Table 2 presents the consumers’ WTP and evaluation of the sensory attributes for each of the four apple samples and the *t*-tests analyzing differences among them. Two sets of *t*-tests were performed. First, differences between apples grown at high and low altitudes for each of the apple cultivars, and second, differences between apple cultivars (‘Golden D.’ and ‘Reineta’) for each of the growing altitudes (last two rows). Results for these *t*-tests indicated that the WTP for the apples grown at high altitudes were statistically higher than for the apples grown at low altitude for both apple cultivars. As mentioned in the introduction, as far as we know, no previous evidence on the difference in WTP between apples grown at high and low altitudes exists, and our results indicate that apples grown at high altitudes could be sold at higher prices than the apples grown in low altitude regardless of the apple cultivar. In addition, the WTP for ‘Golden D.’ was statistically higher than for ‘Reineta’, regardless of the growing altitude, confirming that WTP for apples differs by cultivar as previously stated by [6,8,14,24]. However, whereas Dinis et al. [8] found that ‘Golden D.’ was the least value cultivar and the highest was the local traditional ‘Bravo’ cultivar, our results indicated that consumers were willing to pay less for the traditional apple (‘Reineta’) than for the mainstream variety (‘Golden D.’).

The results of the *t*-tests showed significant differences for all the sensory attributes analyzed by consumers (external appearance, firmness, juiciness, mealiness, sweetness) between apples grown at high and low altitudes for both cultivars, except for the sweetness in ‘Golden D.’ and the juiciness in ‘Reineta’. This result is in line with Charles et al. [11] and Cantín and Gracia [12], who concluded that statistically significant differences in the consumers’ sensory evaluation of apples grown at high and low altitude exist. On the contrary, Mendes da Silva et al. [13]. did not find significant differences in apple sensory characteristics between ‘Gala’ apples grown at different altitudes using both a trained panel and consumers’ evaluation. In the case of ‘Golden D.’ consumers reported a higher rate on external appearance, firmness, juiciness, and sweetness for the apples grown at high altitude than for those cultivated at low altitude, while the contrary was observed for mealiness. These results differ from those found by Charles et al. [11] who concluded that apples from low altitudes were juicer and sweeter than apples from high altitudes. However, it is important to note that they used a trained panel for the evaluation of the sensory attributes instead of regular apple consumers, as we did. In the case of the local cultivar ‘Reineta’, consumers rated firmness lower for the fruit grown at high altitudes, while the contrary was observed for the rest of the sensory attributes, except for juiciness, where no statistical differences were found. On the other hand, statistically significant differences between both cultivars grown at high altitudes were detected for all the sensory attributes and while mealiness was higher in ‘Reineta’ than in ‘Golden D.’, the rest of the sensory properties were higher in ‘Golden D.’. For the apples cultivated at low altitudes, positive statistically significant differences were found for external appearance, juiciness, and sweetness, indicating that ‘Golden D.’ attained higher scores by consumers. All previous results corroborate our first hypothesis that the WTP and the consumers’ sensory attributes perception differ among the samples of the two apple cultivars grown at two different altitudes.

To check our third hypothesis, which is that the effect of sensory attributes on the WTP is different among different apple samples, we conducted a covariance analysis using the Likelihood Ratio test (LR). As mentioned above, Equation (2) is specified using as endogenous variable the WTP pooled for the four apples and as exogenous, the sensory attributes (external appearance, firmness, juiciness, mealiness, and sweetness), the three apple dummy variables (DGolHigh, DGolLow, and DReiHigh) and these variables interacting with the sensory attributes’ variables. Thus, different model specifications are estimated, and the corresponding null hypotheses are tested using an LR test (Table 3). First, we specified the restricted model with only the sensory attributes variables as explanatory variables and the whole model where we also included the apple dummies and these apple dummies interacting with the sensory attributes. The LR test between these two models accounted for 49.94, which is higher than the chi-square for 18 degrees of freedom and a 5% significance level (28.86). Thus, the null hypothesis was rejected, indicating that statistically significant differences across the four apple samples existed. In addition, when we conducted the test between the whole model and the model with the sensory attributes and the apple dummies (only the apple dummies model), results indicated that the null hypothesis is rejected because the LR accounts for 40.4, which is higher than the chi-square of 13 degrees of freedom (22.36) (Table 3). This means that apple dummies’ interactions with the sensory attributes were statistically different across apple samples. Finally, when we compared the whole model and the model with the sensory attributes and the apple dummies interacting with the sensory attributes (only the apple dummies interaction model), the results indicated that the null hypothesis is rejected (LR accounts for 18.48 that is higher than the chi-square of 3 degrees of freedom (7.81)). Consequently, the best specification for the model is to estimate the WTP for the four apple samples using separated specification models. This result confirmed our second hypothesis, which is that the effect of sensory attributes on the WTP also differs among apple cultivars and growing altitudes. Previous studies have also searched for differences in the effect of sensory attributes on the WTP for apples. However, except for Hong et al. [6], who conducted an F-test to check whether conducting separate regressions for each apple is better than using a single regression with pooled data similar to what we did, the rest only included in the regression a dummy variable for the apples [8,10]. Hong et al. [6] also concluded that separate regressions for each apple are a better specification than a single regression with pooled data.

Table 4 presents the Tobit model for the relation between the WTP and the sensory attributes plus the socio-demographic characteristics for each of the four apple samples. We included these socio-demographic characteristics to investigate our third hypothesis. First, we can conclude that mealiness was the only sensory attribute that did not influence the WTP for the four apple samples because the estimated parameters for this sensory trait were not statistically significant for any of them.

The estimated parameters for the sensory scores of firmness, juiciness, and sweetness in ‘Golden D.’ cultivated at high altitude were statistically significant at a 5% significant level. While the estimated coefficients were positive in the case of juiciness and sweetness, it was negative for firmness. These findings indicated that, as consumers perceived this apple as juicer and sweeter, they were more willing to pay for it. In the case of ‘Golden D.’ cultivated at low altitude, the external appearance and the firmness were statistically significant, and the latter was also negative as in ‘Golden D.’ cultivated at high altitude. On the other hand, the estimated coefficient for external appearance was positive, indicating that the external appearance of the apple had a positive impact on the WTP. The findings for firmness indicated that consumers who perceived ‘Golden D.’ as firmer were less willing to pay for it, regardless of the growing place. In the case of ‘Reineta’ cultivated at high altitude, the estimated parameters for the external appearance, juiciness, and sweetness were positive and statistically significant at a 5% significance level, indicating that as consumers perceived the ‘Reineta’ cultivated at high altitude as having a better external appearance and being juicer and sweeter, they were more willing to pay for it. For ‘Reineta’ cultivated at low altitude, estimated coefficients for firmness, juiciness, and sweetness were positive and statistically significant. Then, as consumers perceived the ‘Reineta’ apples cultivated at low altitude as a juicer and sweeter, they were more willing to pay for them. In addition, in the case of ‘Reineta’ cultivated at low altitude, as consumers perceived this apple as firmer, they were more willing to pay, while the contrary was observed for ‘Golden D.’, regardless of the growing altitude. In general, we can conclude that juiciness and sweetness positively influence the consumers’ WTP for the analyzed apple fruits except for ‘Golden D.’ grown at low altitudes. Similar results were found by Bi et al. [25] for tangerines in the US market. In addition, another important finding is that the effect of the sensory attributes’ perception on the WTP differs across apple cultivars. The same results were also found by Hong et al. [6], who studied the effect of external appearance, firmness, juiciness, and sweetness on the WTP for ‘Delicia’, ‘Royal Gala’, and ‘Fuji’ apples. They found that external appearance and sweetness were not statistically significant to explain the WTP for the three apple cultivars, while firmness positively influenced the WTP for ‘Royal Gala’ but did not impact the WTP for ‘Delicia’ and ‘Fuji’. In addition, juiciness had a negative impact on the WTP for ‘Delicia’, positive for ‘Royal Gala’, and no effect for ‘Fuji’. Other papers also added some evidence to the effect of some sensory attributes on the WTP for different apple cultivars. McCluskey et al. [7] and McCluskey et al. [9] concluded that firmness and sweetness had a positive impact on the WTP for ‘Gala’ and ‘Red Delicious’ apples. The same result was found by Gallardo et al. [10] for ‘Gala’ and ‘Honeycrisp’.

Finally, a few consumers’ socio-demographic characteristics influenced the WTP for the four apple samples. The estimated parameter for ‘elementary education’ was statistically significant and negative for ‘Golden D’ grown at a high altitude at a 5% significance level, indicating that consumers with only elementary school were less willing to pay for it. None of the socio-demographic characteristics was statistically significant for ‘Golden D.’ cultivated at low altitude. For the two ‘Reineta’ samples, the estimated parameter for the variables ‘female’ and ‘high income’ were statistically significant, although positive for the latter and negative for the former. These results indicate that women were less willing to pay for the two ‘Reineta’ apple samples, while higher-income consumers were more willing to pay for them. In addition, we can conclude that consumers’ age did not explain the WTP for any of the four apple samples, while the impact of the rest of the socio-demographic depended on the apple cultivar and the growing altitude. These results corroborated to some extent our third hypothesis, which claims that consumers’ socio-demographic characteristics influence their WTP for the different apple samples, since gender and income consistently explain the WTP for the ‘Reineta’ cultivar, while the consumers’ age had no impact on the WTP for any of the four analyzed samples. The latter results contradicted previous studies that included the socio-demographic in the explanation of the WTP [7,9,10] because they found that age, as well as ethnicity and employment, explained the WTP in some cases.

## 4. Conclusions

The WTP for ‘Golden D.’ was higher than for ‘Reineta’, and higher for the apples grown at high altitudes than at low altitudes. The perception of all the sensory attributes studied was different between the apples grown at high and low altitudes regardless of the apple cultivar (except for sweetness in ‘Golden D.’ and juiciness in ‘Reineta’). In addition, the perception of all the sensory attributes for the apples cultivated at high altitudes differed between both cultivars, while only the external appearance, the juiciness, and the sweetness were different between cultivars grown at low altitudes.

Our results have clear implications for apple producers and marketers since they provide information about the impacts of external and internal attributes on consumers’ acceptance and willingness to pay (WTP), which is a valuable tool for designing marketing strategies. They also provide information about which are the main quality traits that influence consumers’ WTP. Therefore, our results will help producers and marketers to identify the best cultivars of fresh apples to be introduced into the market.

External appearance only positively influenced the WTP for ‘Golden D.’ at low altitude and ‘Reineta’ at high altitude, although the external appearance was always higher for both apple cultivars when grown at high altitude. The only internal sensory property that did not influence the WTP for the four apple samples was mealiness. Juiciness and sweetness positively influenced the WTP for all the apple samples, except for ‘Golden D’ grown at low altitudes, where no effect of these attributes on the WTP was detected. Firmness negatively influenced the WTP for ‘Golden D.’ cultivar, while a positive effect was observed for ‘Reineta’, but only when cultivated at low altitude. These findings indicate that sweeter and juicer apples might be marketed at higher prices and that firmness is only valued in some cases.

Consumers with only an elementary degree were less willing to pay for ‘Golden D.’ cultivated at high altitudes. In addition, male consumers with higher income levels were more willing to pay for both ‘Reineta’ samples, regardless of the cultivated area.

In summary, our results highlight the clear impact of the growing altitude on the consumer perception of apple quality and on their WTP. In addition, they indicate that WTP is highly influenced by the sensory experience of the apple in a previous purchase experience, and therefore shows that apple eating experience at the first purchase determines subsequent purchases and WTP.

Our results indicate that the internal attributes that consistently support higher pricing of the apples in the market are sweetness and juiciness. On the other hand, mealiness has no impact on apple prices, while firmness negatively affects the price of the ‘Golden D.’ cultivar, probably due to excessive firmness. Finally, external appearance only supports higher prices for two of the four analyzed apple samples in this work. On the other hand, our results also indicate that higher-income consumers are more willing to pay higher prices for local apples, which is an important result to take into account when designing marketing strategies for local fruit products.

## Figures and Tables

**Table 1:** Socio-demographic characteristics of apple consumers population sample.

Characteristics	Sample (*n* = 195)	Population †
Gender		
Female	60.0	51.71 ^1^
Male	40.0	48.31 ^1^
Age (average, standard dev)	50.6 ± 17.0	44.52 ^2^
18–34	21.0	20.61 ^1^
35–44	13.9	18.41 ^1^
45–54	23.1	18.71 ^1^
55–64	20.0	17.21 ^1^
≥65	22.0	25.11 ^1^
Studies level		
Primary	26.7	34.33 ^3^
Secondary	26.1	26.53 ^3^
Higher	47.2	39.53 ^3^
Income range		
≤1500 EUR/month	30.3	n.a.
1501–2500 EUR/month	18.5	n.a.
2501–3500 EUR/month	16.9	n.a.
>3500 EUR/month	8.7	n.a.
Do not know/refuse to answer	25.6	n.a.
Family size (average, standard dev)	2.8 ± 1.1	n.a.
Children less than 18 years old		
0	69.0	n.a.
1	18.3	n.a.
2	11.3	n.a.
3	1.6	n.a.
Vegetarian	5.7	n.a.
Living in the region for more than 20 years (%)	86.0	n.a.
Frequency of food shopping		
Always	46.2	n.a.
Often	33.3	n.a.
Number of fresh fruits eaten daily (average, standard dev)	2.6 ± 1.2	n.a.

^1^ [21]; ^2^ [22]; ^3^ [23]; † Province where the town is located; Note: the % of respondents is presented, unless otherwise stated.

**Table 2:** Summary statistics and paired *t*-test values for pairwise comparisons of WTP and sensory attributes scores by growing altitude and apple cultivar.

	WTP		Sensory Attributes				
	Mean	% of Zero WTPs	External Appearance	Firmness	Juiciness	Mealiness	Sweetness
‘Golden D.’							
High altitude	2.10 (0.69)	0.51	6.90 (0.12)	7.34 (0.12)	7.43 (0.11)	3.70 (0.15)	6.47 (0.14)
Low altitude	1.65 (0.63)	3.1	6.03 (0.11)	6.06 (0.13)	6.08 (0.12)	4.73 (0.16)	6.18 (0.14)
*t*-test	9.05 ***		6.39 ***	8.18 ***	9.27 ***	−5.97 ***	1.72
‘Reineta’							
High altitude	1.62 (0.98)	16.4	6.14 (0.12)	4.67 (0.18)	5.71 (0.16)	6.61 (0.16)	4.77 (0.18)
Low altitude	1.42 (0.85)	16.4	5.51 (0.12)	6.36 (0.16)	5.48 (0.15)	4.69 (0.17)	3.69 (0.16)
*t*-test	3.18 ***		4.76 ***	−8.18 ***	1.21	9.71 ***	5.92 ***
High altitude: ‘Golden D.’ versus ‘Reineta’							
*t*-test	6.89 ***		5.42 ***	13.87 ***	9.99 ***	−13.81 ***	9.10 ***
Low altitude: ‘Golden D.’ versus ‘Reineta’							
*t*-test	3.63 ***		3.41 ***	−1.26	3.35 ***	0.095	12.61 ***

*** denote statistical significance at 0.001 significance levels. Note: Standard deviations are in parentheses.

**Table 3:** Likelihood Ratio test for testing whether the effect of the sensory attributes on the WTP is different among apple varieties and growing altitude.

	Log Likelihood	LR	d.f.
Restricted model: sensory attributes	−932.22		
Whole model: sensory attributes + apple dummies + apples dummies interacting sensory attributes	−907.25		
Test: H0: All apple dummies + apple dummies interacting with sensory attributes coefficients = 0 H1: Otherwise		49.94	18
Only apple dummies model: sensory attributes + apple dummies	−927.45		
Test: H0: All apple dummies interacting with sensory attributes coefficients = 0 H1: Otherwise		40.4	13
Only apple dummies interaction model: sensory attributes + apples dummies interacting sensory attributes	−916.49		
Test: H0: All apple dummies coefficients = 0 H1: Otherwise		18.48	3

Note: Apple dummy variables: DGolHigh (1 for the ‘Golden D.’ apple cultivated at higher altitude and 0 otherwise); DGolLow (1 for the ‘Golden D.’ apple cultivated at lower altitude and 0 otherwise); and DReiHigh (1 for the ‘Reineta’ apple cultivated in higher altitude and 0 otherwise). ‘Reineta’ apple cultivated at higher altitude was dropped to avoid multicollinearity. Apple dummy variables interacting with sensory attributes, for example, DGolHigh*firmness or DGolLow*sweetness, and so on, defining 3 × 5 apple dummies interactions.

**Table 4:** Effects of sensory attributes scores and consumers’ socio-demographic characteristics in the WTP for the apples: estimations of the Tobit model.

	‘Golden D.’ High	‘Golden D.’ Low	‘Reineta’ High	‘Reineta’ Low
Constant	1.8057 (5.25) ***	1.4065 (4.05) ***	−0.2209 (−0.54)	0.2554 (0.71)
Sensory attributes				
External appearance	−0.0193 (−0.51)	0.0883 (2.39) **	0.1996 (4.48) ***	0.0418 (0.92)
Firmness	−0.0750 (−2.44) **	−0.0708 (−1.79) *	0.0483 (1.51)	0.0630 (1.81) *
Juiciness	0.0826 (2.47) **	0.0016 (0.04)	0.0996 (2.19) **	0.0732 (1.76) *
Mealiness	−0.0311 (−1.36)	−0.0162 (−0.87)	−0.0398 (−1.18)	−0.0399 (−1.32)
Sweetness	0.0914 (3.31) ***	0.0238 (0.94)	0.1000 (3.11) ***	0.0942 (3.12) ***
Socio-demographics				
Years (continuous)	−0.0137 (−0.39)	0.0022 (0.64)	−0.00725 (−1.26)	0.0017 (0.38)
Female (dummy)	0.0564 (0.59)	−0.0004 (−0.00)	−0.2388 (−1.80) *	−0.3700 (−2.78) **
High income (dummy)	0.0067 (0.04)	−0.1781 (1.06)	0.5911 (2.14) **	0.5220 (2.07) **
Elementary education (dummy)	−0.3027 (−2.47) **	−0.0485 (−0.38)	−0.0694 (−0.40)	0.0417 (0.24)

***, **, and * denote statistical significance at 1%, 5%, and 10% significance levels, respectively.

## Data Availability

The data presented in this study are available on request from the corresponding author.
